# DcaP-Family Porins are Required for Carboxylic Acid Catabolism in *Acinetobacter baumannii*

**DOI:** 10.1101/2025.07.02.662759

**Published:** 2025-07-15

**Authors:** Hannah R. Noel, Jonathan D. Winkelman, Lauren D. Palmer

**Affiliations:** 1 Department of Microbiology and Immunology, University of Illinois Chicago, Chicago, IL, USA; 2 Trestle LLC, Milwaukee, WI, USA

## Abstract

*Acinetobacter baumannii* is a pathogen of concern and a leading cause of multidrug-resistant healthcare-associated infections. The *A. baumannii* outer membrane is a barrier to antimicrobials and host defenses but must also allow essential nutrients to permeate. Here, we investigate the functional importance of the putative DcaP-family outer membrane porins, which include a proposed vaccine target in *A. baumannii*. All *A. baumannii* genomes surveyed encode multiple DcaP family porins, which we classify in four classes based on protein sequence phylogeny (DcaP1–4). DcaP proteins encoded by species in other genera could not be mapped to these DcaP classes and phylogenetic analysis suggests DcaP1–4 proteins diversified within *Acinetobacter*. Phenotypic array assays and additional experiments show that DcaP3 was necessary for growth on multiple di- and tri-carboxylic acids as sole carbon sources, including citric acid and tricarballylic acid. Finally, a mutant lacking all DcaP proteins was attenuated in the liver and spleen in a mouse model of bloodstream infection and complemented by expression of DcaP3. These findings provide insight on how *A. baumannii* acquires nutrients through the outer membrane barrier and show DcaP3 is important during infection in specific host niches, validating its potential as a therapeutic target.

## INTRODUCTION

*Acinetobacter baumannii* is a pathogen of critical concern and a major cause of healthcare-associated infections. Isolates of *A. baumannii* are often multidrug resistant (MDR) or extensively drug resistant (XDR) and exhibit resistance to first and last line antibiotics, such as meropenem and colistin, respectively (1). The Centers for Disease Control and the World Health Organization have therefore identified *A. baumannii* as an urgent public health threat, calling for new avenues of treatment (2–4). The bacterial cell envelope is the primary barrier from the environment and protects bacteria from environmental stress including antibiotics and host defenses; however, the cell envelope must also allow acquisition of essential nutrients and cofactors (5,6). The outer membrane (OM) of *A. baumannii* is thought to be less permeable compared to other Gram-negative bacteria such as *E. coli*, contributing to intrinsic antibiotic resistance (7). How small molecule nutrients required for growth transit the impermeable OM in *A. baumannii* is incompletely understood.

The OM of *A. baumannii* is an asymmetric lipid bilayer where the inner leaflet is composed of glycerophospholipids and the outer leaflet of lipooligosaccharides (LOS) (5,8,9). The OM bilayer is occupied by proteins that serve a variety of functions, including nutrient influx. Nutrients typically pass through the OM via active transport by TonB-dependent transporters or passive diffusion through porins which are transmembrane channels that allow passage of small molecules (6,10). The primary porin in *A. baumannii* is OmpA, which shows slow molecular transport in liposome swelling assays, contributing to the low permeability barrier of the OM (11–13). However, the primary function of OmpA is thought to be structural, providing a physical tether between the OM and the peptidoglycan cell wall in *A. baumannii* and other species (14,15). *A. baumannii* encodes multiple other porins including CarO, an OM porin that selectively allows influx of ornithine and other basic amino acids (16), and the Occ family porins, responsible for the influx of compounds like benzoate, hydroxycinnamate, and other aromatic molecules (17–19). The protein DcaP is a predicted OM porin widely distributed across *Acinetobacter* species named for its localization within a genetic locus for utilization of dicarboxylic acids (*dca*) (20). Notably, a DcaP family protein is among the most abundant OM proteins in *A. baumannii* during rat and mouse infections (21). Due to this fact, multiple groups have studied a DcaP-like protein as a promising vaccine candidate for protection against *A. baumannii* infection (21–25). However, the biological role of DcaP family proteins remains unclear.

First identified in Parke *et al*. as part of the dicarboxylic acid catabolic operon of *Acinetobacter baylyi* ADP1, *dcaP* (dicarboxylic acid porin) and DcaP-like proteins were thought to be specific to the Moraxellaceae family (20). The structure for one DcaP family protein has been solved, presenting as a homo-trimeric 16-stranded beta-barrel protein with an extended periplasmic N-terminus that forms a coiled coil (21). Due to the genetic localization and induction of expression with the dicarboxylic acid adipate, *A. baylyi* DcaP-like proteins were proposed to be nonspecific porins that facilitate the uptake of dicarboxylic acids (20); however, a role for DcaP-like proteins in carboxylic acid catabolism has not been reported. Molecular dynamics and electrophysiology studies determined a DcaP-like protein to have an affinity for anionic compounds as substrates, such as the clinically relevant β-lactamase inhibitor sulbactam (21). In other studies, DcaP-like proteins have been implicated in biofilm formation and mucin catabolism (26,27). Here, we describe four classes of DcaP-like proteins in *Acinetobacter* and investigate their role in carbon source utilization.

## RESULTS

### Acinetobacter spp. encode multiple DcaP-family porins

**Figure 1.**
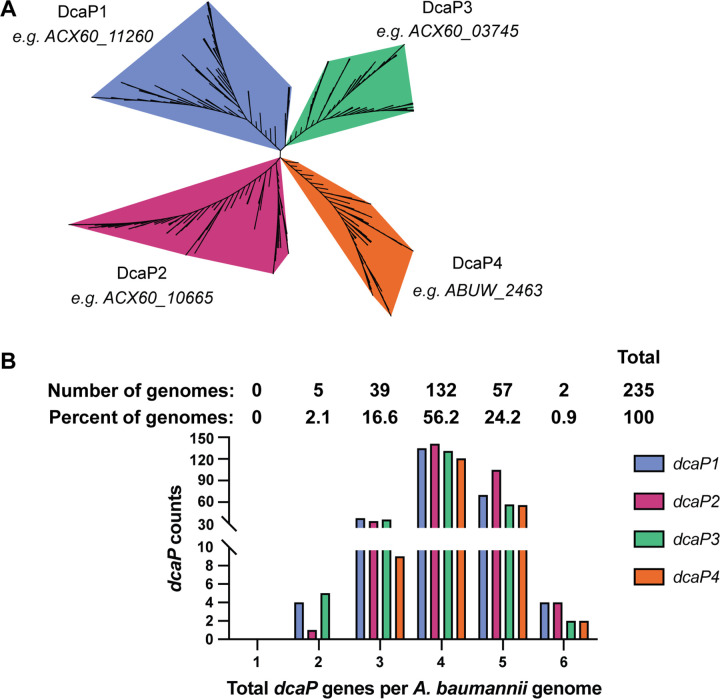
*A. baumannii* DcaP proteins cluster in four classes and are widely distributed among *Acinetobacter*. **(A)** An unrooted tree of DcaP protein sequences from 255 *Acinetobacter* genomes (235 *A. baumannii* genomes). **(B)** Manual counts and distribution of individual DcaP proteins binned by the total number of DcaP proteins in an *A. baumannii* genome.

The first DcaP was described in *A. baylyi* as part of a dicarboxylic acid catabolic operon (20), and DcaP-like proteins have been identified in various *A. baumannii* clinical isolates and type-strains (21,23). Proteins designated DcaP-like in *A. baumannii* ATCC 17978 are homologous to *A. baylyi* DcaP, sharing ~30–35% amino acid identity by Clustal Omega multiple sequence alignment (28). However, given the abundance of DcaP-like proteins within individual *Acinetobacter* genomes, we hypothesized that these proteins could be further classified based on protein sequence. Using DcaP and DcaP-like sequences from a set of deduplicated *A. baumannii* genomes, four distinct classes of DcaP-like proteins were identified that cluster independently, including the original DcaP which we display as DcaP1 (Fig. 1A, S1A). We next determined the distribution of DcaP-like proteins across the *Acinetobacter* genus. Using a set of 250 deduplicated *Acinetobacter* genomes, most of which were *A. baumannii*, DcaP proteins were shown to be widely conserved across the genus (Fig. S1B). Additionally, *Acinetobacter* species within the *A. calcoaceticus/baumannii* (*acb*) complex/clade of pathogenic *Acinetobacter* appeared to be enriched for having multiple DcaP proteins compared to non-pathogenic *Acinetobacter* species (Fig. S1B). Among the *A. baumannii* genomes, over 130 of the strains encode four *dcaP* genes (Fig. 1B). In fact, no *A. baumannii* genome analyzed encoded fewer than two *dcaP* genes (Fig. 1B). To determine if the *dcaP* genes were equally distributed across the species, the number of each DcaP subfamily were tallied for each genome and organized based on how many total DcaP proteins were present. The four classes of DcaP proteins were equally likely to be encoded in a given *A. baumannii* genome (Fig. 1B). For example, in most cases where a genome encodes four DcaP proteins, it was most likely to have one of each variant. Alternatively, in a genome encoding five DcaP proteins, DcaP2 was most likely to be duplicated. These data suggest that DcaP proteins are encoded across the *Acinetobacter* genus, and enriched in pathogenic *Acinetobacter,* including *A. baumannii*.

### DcaP proteins from other bacterial species are distinct from Acinetobacter DcaP proteins

**Figure 2.**
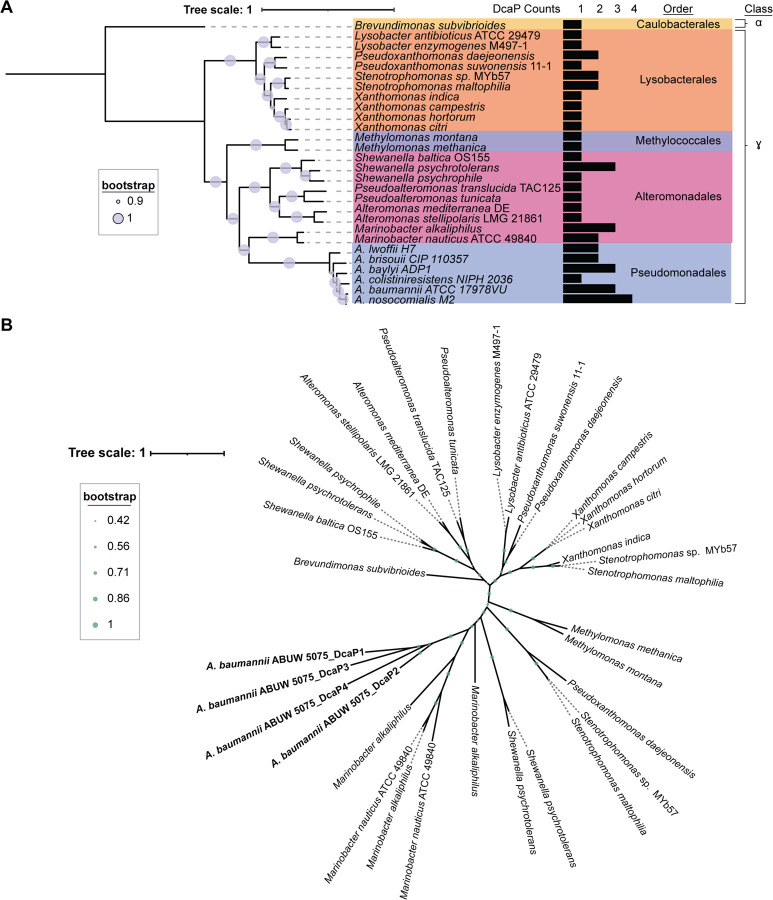
*A. baumannii* DcaP-family proteins cluster together compared to non-*Acinetobacter* DcaP proteins. **(A)** A rooted tree of DcaP containing species outside of *Acinetobacter* and some *Acinetobacter spp.* depicting class, order, and number of encoded DcaP proteins. **(B)** An unrooted tree of DcaP protein sequences from non-*Acinetobacter* species with AB5075 as the *A. baumannii* reference. Tree scale in amino acid substitutions.

To investigate the evolutionary history of DcaP proteins in *Acinetobacter* and determine if DcaP diversified within *Acinetobacter* or across phyla, orthologues in other bacterial phyla were identified through KEGG. Bacterial phyla were selected for further analysis if the DcaP-like protein was encoded in more than half of the genomes for a given genus (29,30). A rooted phylogenomic tree was generated to demonstrate the evolutionary distance between *Acinetobacter* and the bacteria containing a DcaP orthologue (Fig. 2A). Orthologues of DcaP proteins were found in distantly related organisms, such as *Shewanella spp.* and *Xanthomonas spp.,* albeit generally in fewer numbers (Fig. 2A). These DcaP orthologues could not be further classified into one of the four DcaP classes defined in *A. baumannii*. This suggests that the four DcaP classes of proteins defined here are unique to *Acinetobacter spp*. To better understand the relationship between *Acinetobacter* DcaP proteins and proteins designated DcaP-like from distantly related species, an unrooted tree of protein sequences was generated. This analysis revealed that DcaP proteins from AB5075, the included reference *A. baumannii* strain which encodes DcaP1–4, were more similar to each other than to non-*Acinetobacter* DcaP proteins (Fig. 2B). This suggests that diversification of the four DcaP classes defined here occurred within *Acinetobacter*. In contrast to *A. baumannii*, multiple DcaP-like proteins encoded in other organisms were sequence divergent, such as in *Pseudoxanthomonas daejeonensis*, *Stenotrophomonas spp*., *Shewanella psychrotolerans*, and *Marinobacter spp.* (Fig 2B). Comparison of the species phylogeny and DcaP protein phylogeny suggest there may have been occurrences of horizontal gene transfer in other species outside *Acinetobacter*. Overall, these data suggest that DcaP-like proteins are encoded by species across Proteobacteria and that DcaP diversification has occurred within the *Acinetobacter* clade.

### DcaP family porins are necessary for growth on select di- and tri-carboxylic acids

Next, we hypothesized that the DcaP-family porins are involved in small molecule uptake. One study suggested that DcaP3 may translocate the clinically relevant beta-lactamase inhibitor sulbactam based on electrophysiology and applied field simulation data (21). Sulbactam is a therapeutic often used in combination with beta-lactam antibiotics like ampicillin for bacteria with beta-lactam resistance. Many *A. baumannii* isolates are intrinsically resistant to ampicillin due to the expression of beta-lactamases and sulbactam has been shown to resensitize resistant isolates to beta-lactam antibiotics (31,32). We reasoned that if DcaP proteins facilitated sulbactam entry, a ΔΔΔ*dcaP* mutant would exclude sulbactam from entering the cell and result increased resistance to the beta-lactam ampicillin while a wild-type strain would exhibit a re-sensitization to ampicillin. However, the wild-type and the ΔΔΔ*dcaP* mutant strains exhibited similar sensitivities to ampicillin/sulbactam treatment (Fig. S2). These data suggest that DcaP proteins are not required for sulbactam activity.

**Figure 3.**
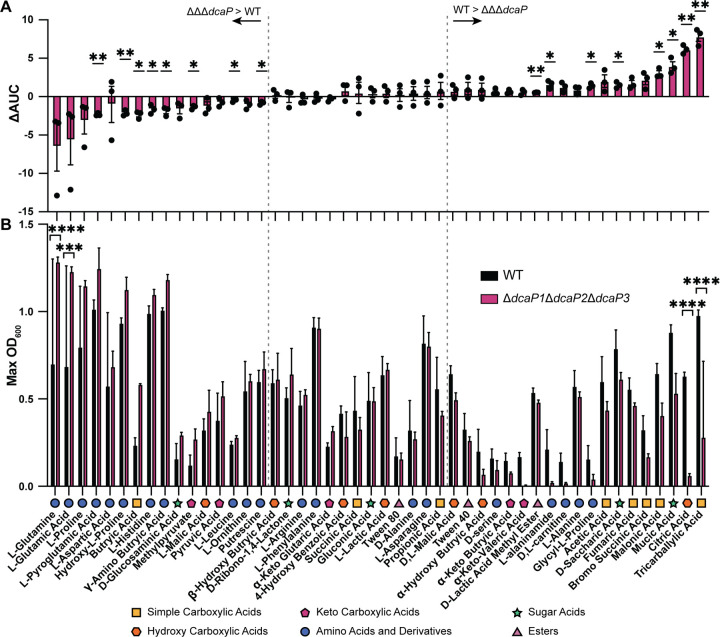
Wild-type *A. baumannii* grows better than ∆∆∆*dcaP* mutant on certain di- and tricarboxylic acids as sole carbon sources. Biolog plates PM1 and PM2a were resuspended in M9 minimal media without a carbon source and inoculated with either WT or the **∆∆∆***dcaP* mutant. (**A**) Difference in area under the curve (ΔAUC) was calculated for WT – **∆∆∆***dcaP* mutant. Each point is from one experiment with 1 biological replicate per strain. Significance is by one sample t-test compared to 0. (**B**) Maximum optical density at 600 nm (OD_600_) is shown. Significance is by two-way ANOVA with Sidak’s multiple comparisons. Experiments were conducted three times with an n=1 for a total of n=3. Data are mean +/− SD. *P < 0.05, **P < 0.01, ***P < 0.001, ****P < 0.0001

We next hypothesized that DcaP porins may be important for growth on certain nutrient sources such as dicarboxylic acids as previously suggested (20). The growth of wild-type and ∆∆∆*dcaP* strains were compared using the high-throughput Biolog PM system to screen for carbon source usage. Multiple compounds were identified on which wildtype was able to grow but ΔΔΔ*dcaP* had a defect, and compounds where ΔΔΔ*dcaP* grew more than wildtype (Fig. 3A–B). Many of the compounds in which WT grew better than ΔΔΔ*dcaP* were carboxylic acids, including simple carboxylic acids, hydroxy carboxylic acids, and sugar acids (Fig. 3A–B). Conversely, compounds in which the mutant outgrew WT were largely amino acids and their derivatives (Fig. 3A–B). These data suggest that DcaP porins may allow the influx of carboxylic acid-containing compounds, as previously predicted (20).

### DcaP3 is important for growth on multiple carboxylic acid carbon sources

**Figure 4.**
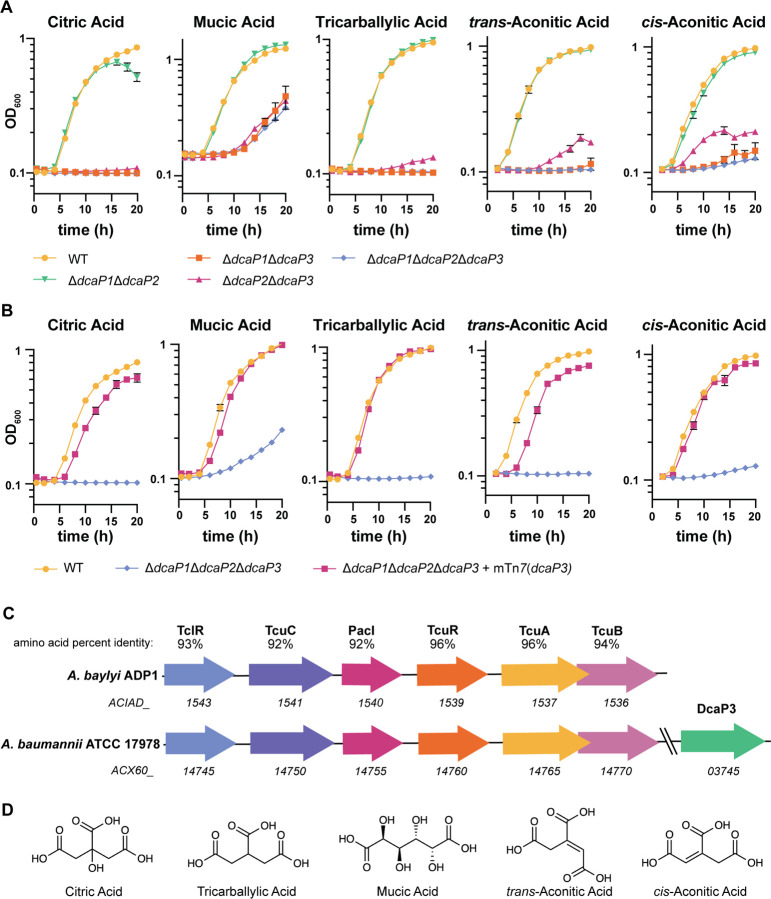
DcaP3 is important for growth on the carboxylic acid carbon sources citric acid, tricarballylic acid, mucic acid, and *cis*- and *trans*-aconitic acid. **(A)** Wildtype, double, and the triple **∆∆∆***dcaP* mutants were grown in M9 media with the indicated compound as the sole carbon source. Optical density at 600 nm (OD_600_) is shown. **(B)** Wildtype, the **∆∆∆***dcaP* mutant, and the **∆∆∆***dcaP* mutant complemented with *dcaP3* were grown on the indicated compound as the sole carbon source. **(C)** The genetic organization of the *tcu* metabolic locus and *dcaP3* in *A. baumannii* ATCC 17978. **(D)** The chemical structures of the relevant carbon source compounds. Data are mean +/− SEM and n=3. Experiments were repeated twice with similar results.

To verify the Biolog results and further determine specificity of the DcaP classes, growth curves using ΔΔΔ*dcaP,* Δ*dcaP1*Δ*dcaP2*, Δ*dcaP1*Δ*dcaP3*, and Δ*dcaP2*Δ*dcaP3* and the carboxylic acids as sole carbon sources were performed. Mutants lacking DcaP3 were defective for growth on citric acid, tricarballylic acid, and mucic acid compared to the wild-type strain (Fig. 4A). We were unable to identify conditions in which DcaP1 or DcaP2 were required for growth. Complementation experiments showed that DcaP3 expression was sufficient to restore growth on these substrates to near wild-type levels (Fig. 4B). Similarly, a single ∆*dcaP3* mutation conferred a defect in growth on citric acid, tricarballylic acid, and mucic acid, whereas single mutants of ∆*dcaP1* or ∆*dcaP2* showed no defect (Fig. S3).

A recent study in *A. baylyi* found that tricarballylic acid metabolism is closely linked with the metabolism of *cis*- and *trans*-aconitic acid (33). The genetic organization of the tricarballylic acid catabolic genes are conserved in *A. baumannii* compared to *A. baylyi* (Fig. 4C). Since the data thus far suggest DcaP3 is necessary for growth on tricarballylic acid, we hypothesized that DcaP3 may also be important for *cis*- and *trans*-aconitic acid growth. Mutants lacking DcaP3 had a defect for growth on both *cis*- and *trans*-aconitic acid (Fig. 4A). Complementing DcaP3 in the chromosome of the triple mutant ∆∆∆*dcaP* restored growth to near wild-type-like levels, confirming the role of DcaP3 in growth on these carbon sources (Fig. 4B). Finally, the ∆*dcaP3* single mutant had a defect in growth on both *cis*- and *trans*-aconitic acid compared to wildtype (Fig. S3). To highlight the similarities in the carbon sources in which DcaP3 is important for growth, the chemical structures for each carbon source are outlined in Fig. 4D. Taken together, these data suggest that DcaP3 is important for growth using multiple di- and tri-carboxylic acids as sole carbon sources.

### DcaP4 complements ΔdcaP3 for growth on carboxylic acids

**Figure 5.**
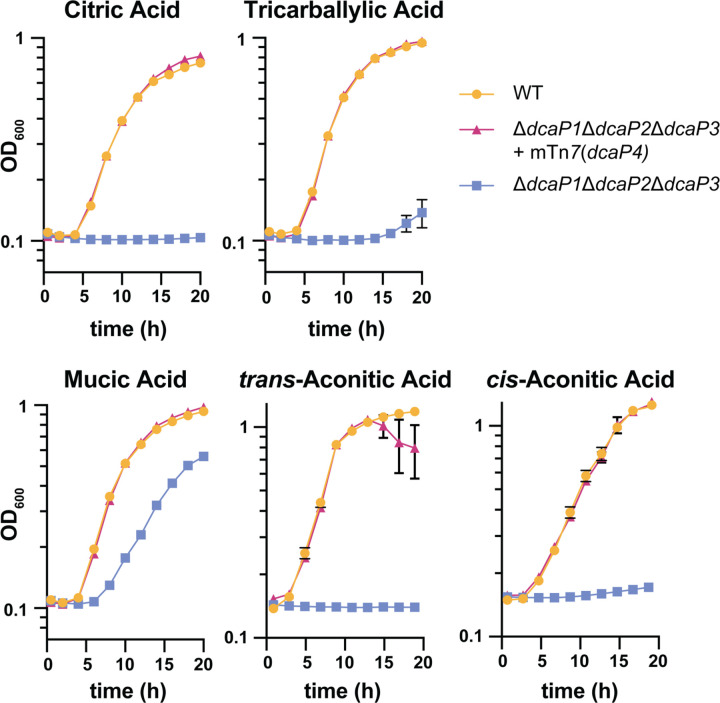
*A. baumannii* AB5075 DcaP4 complements growth of *A. baumannii* ATCC 17978 Δ*dcaP1–3* on citric acid, tricarballylic acid, mucic acid, and *cis*- and *trans*-aconitic acid. Wildtype, the **∆∆∆***dcaP* mutant, and the **∆∆∆***dcaP* mutant complemented with *dcaP4* were grown in M9 media with the indicated compound as the sole carbon source. Optical density at 600 nm (OD_600_) is shown. Data are mean +/− SEM and n=3. Experiments were repeated twice with similar results.

Thus far, experiments were performed with *A. baumannii* ATCC 17978 which does not encode DcaP4; thus, the potential role for DcaP4 in growth using carboxylic acids was unclear. *A. baylyi* can grow on citric acid and tricarballylic acid and does not encode DcaP3 but rather DcaP1, DcaP2, and DcaP4 (33). We hypothesized that DcaP4 could be also allow influx of carboxylic acids. To test this, the DcaP4 from *A. baumannii* AB5075, which shares 77% amino acid identity to *A. baylyi* DcaP4, was expressed from the 17978 DcaP3 promoter in the ∆∆∆*dcaP A. baumannii* mutant. Consistent with previous observations, the ΔΔΔ*dcaP* mutant was unable to grow using citric acid, tricarballylic acid, mucic acid, or *cis*- and *trans*-aconitic acid (Fig. 5). Growth was restored to the ΔΔΔ*dcaP* mutant by expressing DcaP4 chromosomally from the DcaP3 promoter, suggesting that DcaP4 and DcaP3 have overlapping functions in some *A. baumannii* strains (Fig. 5).

### DcaP3 is required for growth on di- and tri-carboxylic acids in a clinical isolate

**Figure 6.**
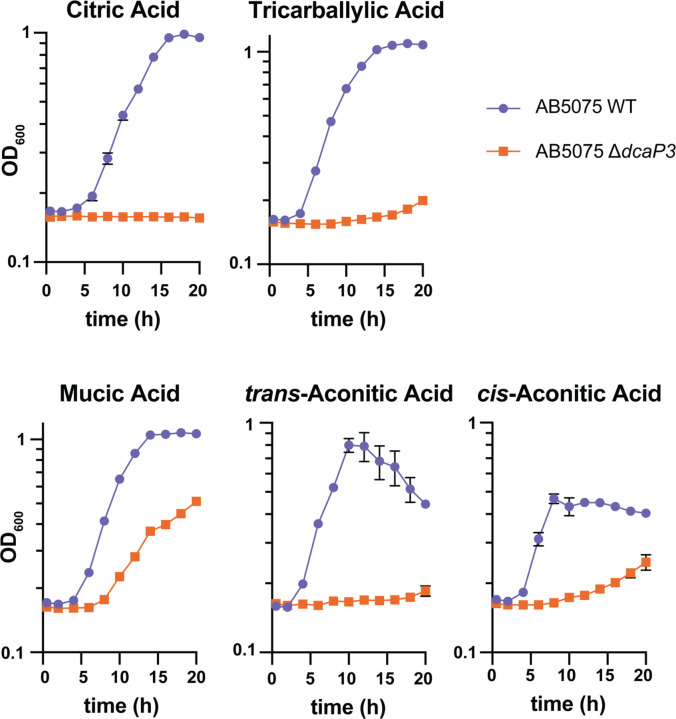
*A. baumannii* AB5075 Δ*dcaP3* is defective for growth on citric acid, tricarballylic acid, mucic acid, and *cis*- and *trans*-aconitic acid. *A. baumannii* AB5075 wild-type and **∆***dcaP3* mutant strains were grown in M9 media with the indicated compound as the sole carbon source. Optical density at 600 nm (OD_600_) is shown. Data are mean +/− SEM and n=3. Experiments were repeated twice with similar results.

To test if DcaP3 is important for growth on di- and tri-carboxylic acids in a more recent clinical isolate, a ∆*dcaP3* mutant was generated in the more recent clinical isolate AB5075 and similar growth curves were performed (34). Mutants lacking *dcaP3* were defective for growth on citric acid, tricarballylic acid, mucic acid, and *cis*- and *trans*-aconitic acid compared to wild-type (Fig. 6). This suggests that the requirement for DcaP3 for growth on select carboxylic acids as sole carbon sources is conserved among *A. baumannii* isolates.

### DcaP3 is required for virulence in the liver and spleen during bloodstream infection of mice

**Figure 7.**
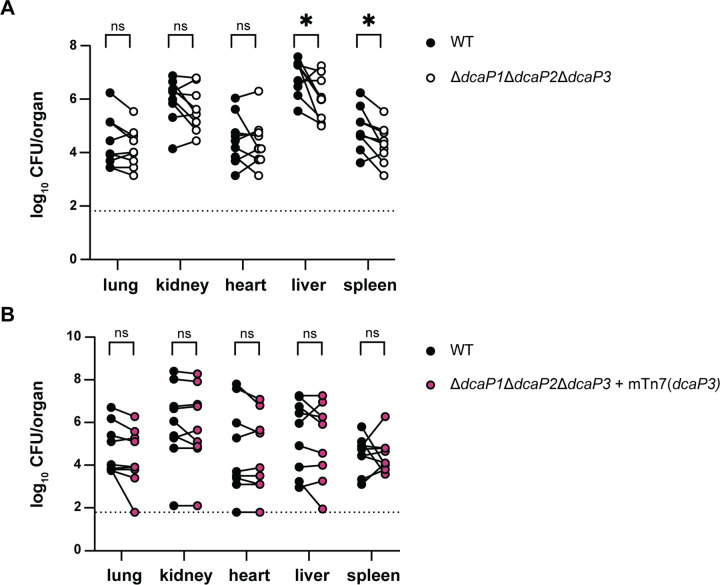
DcaP3 is important for infecting the liver and spleen in a model of bloodstream infection. **(A-B)** Wild-type and the indicated strains were retro-orbitally inoculated into Swiss Webster mice in a 1:1 ratio. At 24 h post-infection, organs were harvested and bacterial CFU were enumerated. The dotted lines indicate the limit of detection. Each point represents one strain enumerated from one mouse; n=9–10. Data are combined from two experiments. Statistics are Wilcoxon non-parametric test. *P < 0.05.

Carbon source utilization can be a critical determinant of pathogenesis for *A. baumannii* and other bacterial species in different host niches (35,36). Therefore, we sought to determine if DcaP function is important for *A. baumannii* pathogenesis. To test this, murine bloodstream infections were performed, co-inoculating wild-type and the ∆∆∆*dcaP* mutant or wild-type and the ∆∆∆*dcaP* mutant complemented with DcaP3. We hypothesized that there may be a host niche in which DcaP proteins are important for *A. baumannii* replication, leading to a defect for ∆∆∆*dcaP* compared to wildtype. There was no defect of the ∆∆∆*dcaP* mutant compared to wildtype in lung, kidney, or heart (Fig. 7A). By contrast, there was a defect for the ∆∆∆*dcaP* mutant compared to wildtype in the spleen and liver (Fig. 7A). The defect in the spleen and liver was reversed by complementing *dcaP3* in the chromosome of the ∆∆∆*dcaP* mutant, showing DcaP3 is important for replication in the liver and spleen (Fig. 7B). Together these data suggest that carboxylic acid permeability by DcaP3 is important in specific host niches during infection.

## DISCUSSION

The OM protects *A. baumannii* from antimicrobials and other host stresses but must permit influx of nutrients essential for replication. How nutrients pass through the *A. baumannii* OM is not well understood. Here, we designate classes of a family of predicted porins, DcaP1–4, that are widely distributed across *Acinetobacter spp.* and show DcaP3 or DcaP4 are required for growth on multiple carboxylic acids.

DcaP-like porins have been identified in genetic screens for biofilm formation, desiccation, and mucin catabolism, largely referred to as DcaP-like proteins without further distinction (26,27,37–39). We show that *A. baumannii* DcaP-like proteins can be grouped into four classes based on protein sequence (Fig. 1A, S1A). The presence of DcaP-encoding genes is conserved among *Acinetobacter* spp, and particularly in *A. baumannii* and the *acb* complex (Fig. 1B, Fig. S1B). While DcaP proteins can be found in distantly related organisms (Fig. 2A), the DcaP1–4 classes we define in *A. baumannii* cluster together (Fig. 2B). These findings suggest DcaP1–4 diversified within the *Acinetobacter* clade.

Antibiotic resistance in *A. baumannii* is a critical public health problem due to the emergence of XDR isolates. Various porins in *A. baumannii* have been implicated in the uptake of toxic molecules including antibiotics (12,40–42). DcaP3 was proposed to allow entry for the beta-lactamase inhibitor sulbactam (21). Here, we show encoding DcaP-like proteins has no impact on sensitivity to ampicillin in the presence of sulbactam under the conditions tested (Fig. S2). These data suggest DcaP-like proteins may not be required for function of sulbactam.

The transport of essential nutrients across the cell envelope is incompletely understood in *A. baumannii*. In general, *A. baumannii* utilizes organic acids and amino acids as carbon sources and cannot utilize sugars such as glucose (35). Accordingly, multiple OM channels have been identified that are important for growth on specific carbon sources including ornithine, arginine, glutamic acid, and benzoic acid (16–19,43). One study in ATCC 19606 found that DcaP1 (*A1S_1380*) was upregulated in the presence of mucin, suggesting DcaP1 may play a role in the influx of mucin components (26). In a Biolog screen, we identified multiple carboxylic acid carbon sources that supported significantly more growth of the wildtype compared to the ∆∆∆*dcaP* mutant (Fig. 3), suggesting DcaP-family proteins are important for their diffusion through the OM.

The data suggest DcaP3 is responsible for the influx of carboxylic acid carbon sources citric acid, mucic acid, tricarballylic acid, and *cis*- and *trans*-aconitic acid in *A. baumannii* ATCC 17978, as mutants lacking DcaP3 show decreased growth on these substrates (Fig. 4A–B, S3). However, the Δ*dcaP3* mutant grew more than the ∆∆∆*dcaP* mutant in ATCC 17978, suggesting DcaP1 or DcaP2 may play a minor role in growth with these carbon sources (Fig. S3). Citric acid is an intermediate of the tricarboxylic acid (TCA) cycle, a ubiquitous pathway for energy generation. Despite this, many bacteria cannot utilize citric acid as a carbon source and how citric acid permeates the OM is unknown (44). Mutants in *P. aeruginosa* OpdH were defective for growth on *cis*-aconitic acid, but not on citric acid, suggesting they encode other citric acid transporters (45,46). A ∆∆∆*dcaP* mutant displayed a strong defect for growth on citric acid that can be rescued by complementing with DcaP3 or DcaP4 (Fig. 4A, 4B, Fig. 5). Therefore, encoding multiple porins for OM citric acid passage may be conserved across bacterial genera.

Mucic acid, or galactaric acid, is a sugar acid derived from the oxidation of galactose and is unrelated to mucin. Mucic acid is not typically found in the host; but, some antibiotic treatments result in increased mucic acid in the human gut (47–49). However, only a subset of *A. baumannii* strains, including ATCC 17978, encode the D-galactaric acid dehydrogenase required for growth on mucic acid (50). The digestive tract is a reservoir for *A. baumannii* colonization that increases the potential for infection and transmission and *A. baumannii* ATCC 17978 can asymptomatically colonize the mouse gut (51–56). Thus, mucic acid may be used as carbon source by some *A. baumannii* in the inflamed gut. Our data suggest that DcaP3 or DcaP4 are important for growth on mucic acid.

The catabolism of *cis*- and *trans*-aconitic acid has been described in *A. baylyi*, and the catabolism of tricarballylic acid in *A. baylyi* and *Salmonella* (33,57,58). However, the OM porin required for growth on these carbon sources in *Acinetobacter* is unknown. Our data suggest that DcaP3 or DcaP4 are important for growth on tricarballylic acid, and *cis*- and *trans*-aconitic acid. Taken together, our data suggests that DcaP3 and DcaP4 are the major porins for passive transport of specific carboxylic acid carbon sources.

The requirement for DcaP3 in growth on select di- and tri-carboxylic acids is conserved in the more recent clinical isolate *A. baumannii* AB5075, as a ∆*dcaP3* mutant has reduced growth in these conditions (Fig. 6). However, this was surprising as *A. baumannii* AB5075 encodes all four DcaP proteins, including DcaP4, which complemented growth in an *A. baumannii* ATCC 17978 ∆∆∆*dcaP* mutant (Fig. 5). We speculate that *dcaP4* may not be expressed under these conditions.

During infection, bacteria must scavenge nutrients from the host to replicate. The *A. baumannii* ATCC 17978 ∆∆∆*dcaP* mutant had a defect compared to wildtype in the mouse liver and spleen that was complemented by expression of *dcaP3* (Fig. 7A–B). This suggests DcaP3 passive import of carboxylic acid carbon sources is important for pathogenesis in the liver and spleen. Alternatively, DcaP3 may play another role in pathogenesis. For example, porins in *A. baumannii* have demonstrated roles for the adhesion or invasion of eukaryotic cells (13,59–62). DcaP3 is the DcaP-like protein that is a proposed vaccine candidate (21–25). These data suggest that neutralizing DcaP3 may also promote *A. baumannii* clearance.

In summary, we further classify a family of porins previously termed DcaP-like into four classes in *Acinetobacter* spp. This classification allows for the differentiation between DcaP-like proteins and the potential to determine individual functions for DcaP1–4 in *Acinetobacter* spp. DcaP3 was critically important for growth on citric acid, tricarballylic acid, mucic acid, and *cis*- and *trans*-aconitic acid as sole carbon sources. A ∆∆∆*dcaP* mutant was attenuated in a competitive murine systemic infection and complemented by expression of DcaP3, suggesting a role for DcaP3 in pathogenesis and validating its potential as a therapeutic target. Our data therefore provides a deeper understanding of how *A. baumannii* acquires nutrients including in the host.

## MATERIALS AND METHODS

### Bacterial strain construction and growth

All bacterial strains and plasmids used in this study are listed in Tables S1 and S2, respectively. The wild-type strain was *A. baumannii* ATCC 17978VU or AB5075 (63). Oligonucleotides are listed in Table S3 and were purchased from Integrated DNA Technologies (Coralville, IA). Strains were grown in Luria-Bertani lysogeny broth (LB; Miller) or on LB with 1.5% (wt/vol) agar unless indicated otherwise. Antibiotics were used at the following concentrations: carbenicillin (Carb), 75 mg/L; kanamycin (Kan), 40 mg/L; chloramphenicol, 15 mg/L; hygromycin (Hyg) 300 mg/L. For deletion constructs, 1000 bp of 3’ and 5’ flanking DNA to the gene of interest was amplified using Q5 High Fidelity 2x Master Mix [New England Biolabs (NEB), Ipswich, MA] from *A. baumannii* ATCC 17978VU or AB5075. The vector pFLP2 was digested with BamHI and KpnI restriction enzymes (NEB, Ipswitch, MA) and gel purified. The kanamycin or hygromycin cassette was amplified from pKD4. Fragments were ligated together using HiFi ligation mix (NEB, Ipswitch, MA), transformed into chemically competent *E. coli* DH5α, and plated to selective media. Transformants were confirmed to contain the insert by PCR. To generate the knockout strains, a triparental conjugation using helper *E. coli* strain HB101 containing pRK2013 was performed. Single-crossover merodiploids containing the integrated pFLP2 vector were chosen based on a Kan^R^ Carb^R^ or Hyg^R^ and sucrose-sensitive (10% w/v; Suc^S^) phenotype. Putative merodiploids displaying were verified by PCR. Merodiploids were then grown on LB agar, resuspended, and plated to LB agar or LB agar with 10% sucrose to select for colonies with the second crossover event. Colonies were then screened for a Kan^R^ Carb^S^ Suc^R^ or Hyg^R^ Suc^S^ phenotype. Mutants were confirmed via PCR.

For chromosomal integration constructs, DNA from ATCC 17978VU or AB5075 was amplified using Q5 High Fidelity 2x Master Mix (NEB, Ipswich, MA). The mTn*7* pKNOCK vector was digested with BamHI and KpnI prior to ligation using HiFi ligation mix (NEB, Ipswitch, MA) and transformation into chemically competent *E. coli* DH5α λ*pir116*^+^. To generate chromosomally integrated mTn7 constructs, a modified four-way parental mating was performed using helper *E. coli* strain HB101 containing pRK2013 and *E. coli* DH5α containing pTNS2 (64). Colonies were screened by PCR for integration into the correct chromosomal locus at the *att* site. All plasmid constructs were verified by whole plasmid sequencing before transforming into *A. baumannii* (Plasmidsaurus, South San Francisco, CA).

### Phylogenetic tree construction

To identify DcaP orthologs and classify them into hierarchical orthogroups (HOGs), OrthoFinder v2.5.5 was used (65). Two datasets were analyzed: (i) a 253-strain dataset including 235 *A. baumannii* strains deduplicated as previously described (66); (ii) a 28-species dataset that included multiple *Acinetobacter* and a broader range of gammaproteobacteria rooted by one alphaproteobacterial. The protein accession numbers and genome accession numbers are listed in Table S4.

Non-Acinetobacter species were selected based on the conservation of DcaP orthologs across the genus or species indicated by KEGG. Specific organisms were selected to portray a representative distribution of DcaP orthologs across bacteria, a group of 22 species. The FastTree v2.1.11-derived (67), manually rooted species tree was used as an input for OrthoFinder HOG inference (65,68), ensuring accurate classification of gene duplication and speciation events. The resulting DcaP classifications were visualized and annotated in iTOL (69). To infer a species phylogeny, a set of 120 universal bacterial marker genes (Bac120) was identified from each proteome using HMMER3 (hmmsearch, v3.3), following the methodology described in Parks *et al*. 2018 (70). The majority of species had only one significant hit for each of the 120 protein HMMs; if a species had more than one hit, the best-scoring hit for that marker was extracted.

Each of the 120 marker proteins was independently aligned to its respective HMM profile using hmmalign (HMMER v3.3). Alignments were trimmed to remove poorly aligned regions and concatenated into a single concatenated supermatrix for each species. To improve alignment quality, columns were filtered based on the following criteria: (i) highly gapped regions (>80% gaps) were removed; (ii) completely homogenous columns were excluded to reduce redundancy.

Maximum likelihood species trees were inferred from the filtered concatenated alignment using FastTree v2.1.11, with the LG model of amino acid evolution. The resulting unrooted trees were manually rooted and annotated in iTOL using *Brevundimonas subvibrioides* or *E.coli* as outgroups for the smaller and larger datasets.

### DcaP protein phylogeny

DcaP homologs were extracted from their respective proteomes by selecting sequences within the DcaP HOG at the root of all *Acinetobacter* species in the 255-species dataset in Orthofinder. To ensure consistent domain identification, sequences were aligned to the Pfam Hidden Markov Model (HMM) PF19577.4, corresponding to the DcaP OM protein family. Alignments were trimmed to remove poorly aligned residues prior to phylogenetic inference. A maximum likelihood phylogenetic tree was constructed using FastTree v2.1.11 with the LG model of amino acid evolution. The inferred clusters were identical to OrthoFinder’s DcaP subfamilies, except for a small cluster of DcaP proteins that OrthoFinder grouped with DcaP1, while FastTree grouped the same cluster with DcaP3.

To identify putative orthologs of DcaP proteins from the 22 species identified above and *A. baumannii* AB5075, proteomes from genomic assemblies were downloaded from the NCBI Datasets database. Two complementary approaches were employed to verify orthology. First, HMMER v3.4 was used to search each proteome with the Pfam profile HMM PF19577, corresponding to the DcaP protein family. Significant hits were retained as candidate DcaP homologs. To independently assess orthology and reconstruct gene relationships, OrthoFinder v2.5.5 was run on all 23 proteomes, which also produced a rooted gene tree for the DcaP family. The resulting DcaP sequences were realigned to the PF19577 HMM using hmmalign, and poorly aligned or unaligned amino acids were trimmed. A maximum likelihood phylogeny was then inferred using RAxML v8.2.12, under the LG amino acid substitution model.

Amino acid sequence alignment and percent identity were determined using the Clustal Omega program (28).

### Carbon source utilization screen using Biolog plates

Plates PM1 and PM2A were obtained from Biolog for carbon source utilization testing. A 110 μL aliquot of defined M9 minimal media lacking carbon was dispensed into every well and resuspended. Then, 100 μL was transferred from the Biolog plates into sterile flat-bottom 96-well plates. Overnight bacterial cultures were diluted 100-fold in sterile PBS before 1 μL of diluted culture was inoculated into every well. Plates were incubated at 37°C with shaking and growth was monitored by optical density at 600 nm (OD_600_) in an EPOCH2 or SynergyH1 BioTek plate reader (Winooski, VT). Background absorbance was subtracted before area under the curve calculations for each isolate and each compound were calculated and the difference between wildtype and ΔΔΔ*dcaP* was analyzed. Compounds were included if any isolate of either wildtype or ΔΔΔ*dcaP* reached an OD_600_ of 0.15 or higher.

### Growth curve analyses

M9 minimal medium without glucose was supplemented 0.1X Vishniac’s trace minerals (71) with the specified carbon source at a concentration to maintain the carbon equivalency of a 4-carbon compound at 16.5 mM. Cultures were inoculated with a single bacterial colony and grown in 3 mL LB media overnight at 37°C with shaking at 180 rpm for 8–16 hours. Growth curves were performed by diluting overnight cultures 100-fold in sterile PBS and inoculating 1 μL diluted culture into 99 μL media in a 96-well flat bottom plate. Growth was monitored as described above.

### Murine bloodstream infection

Six-week-old female ND4 Swiss Webster mice were purchased from Envigo/Harlan Laboratory. Mice were boarded in a temperature-controlled environment with 14:10 h light:dark cycles and food and water were provided as needed. Mice were acclimated to the facility for 1 week prior to infection. Mice were anesthetized with isoflurane and inoculated retro-orbitally with approximately 3 × 10^8^ CFU in a 50 μL bacterial suspension of a 1:1 mixture of *A. baumannii* ATCC 17978VU wildtype and ΔΔΔ*dcaP* mutant or ΔΔΔ*dcaP*+*dcaP3*. For wildtype versus ΔΔΔ*dcaP,* 5 of the mice were infected with wild-type ATCC 17978VU and 5 were infected with ATCC 17978 mTn*7*(Carb^R^). All mice infected with the complement strain were infected with ATCC 17978VU. The inoculum dose was determined by serial dilution and plating on selective agar media. Mice were euthanized at 24 h post infection by CO_2_ asphyxiation, and the organs were excised aseptically. Tissues were homogenized in PBS using a Bullet Blender (Next Advance, Troy, NY), and all samples were serially diluted and plated on LB and LB with carbenicillin or kanamycin selective agar plates for bacterial enumeration. All animal care protocols were approved by the University of Illinois Chicago Institutional Animal Care and Use Committee (IACUC; protocol number 23–119) in accordance with the Animal Care Policies of UIC, the Animal Welfare Act, the National Institutes of Health, and the American Veterinary Medical Association (AVMA). Animals were humanely euthanized consistent with the AVMA guidelines.

### Data reporting, statistical analysis, and figure preparation

Each measurement was taken from a distinct biological sample (e.g., bacterial culture from a single colony or an individual mouse). Data processing and statistical analyses were performed using Microsoft Excel 16.98 and GraphPad Prism 10.4.2. Statistical tests used are indicated in each figure legend. Figures were prepared in Adobe Illustrator 25.2.3.

## Supplementary Material

Supplement 1
