# Supplementary material for: DcaP-Family Porins are Required for Carboxylic Acid Catabolism in Acinetobacter baumannii
Source: bioRxiv. 2025 Jul 15:2025.07.02.662759. Preprint. [Version 2] doi: 10.1101/2025.07.02.662759 (PMC12338516; doi:10.1101/2025.07.02.662759)
Supplement: Supplement 1 [file media-1.pdf]

SI for Noel *et al*., 2025

Supplementary information for

DcaP-Family Porins are Required for Carboxylic Acid Catabolism in *Acinetobacter baumannii*

Hannah R. Noel, Jonathan D. Winkelman, Lauren D. Palmer

Correspondence: ldpalmer@uic.edu

This PDF includes:

Figures S1 to S4

Tables S1 to S3

Supplemental methods

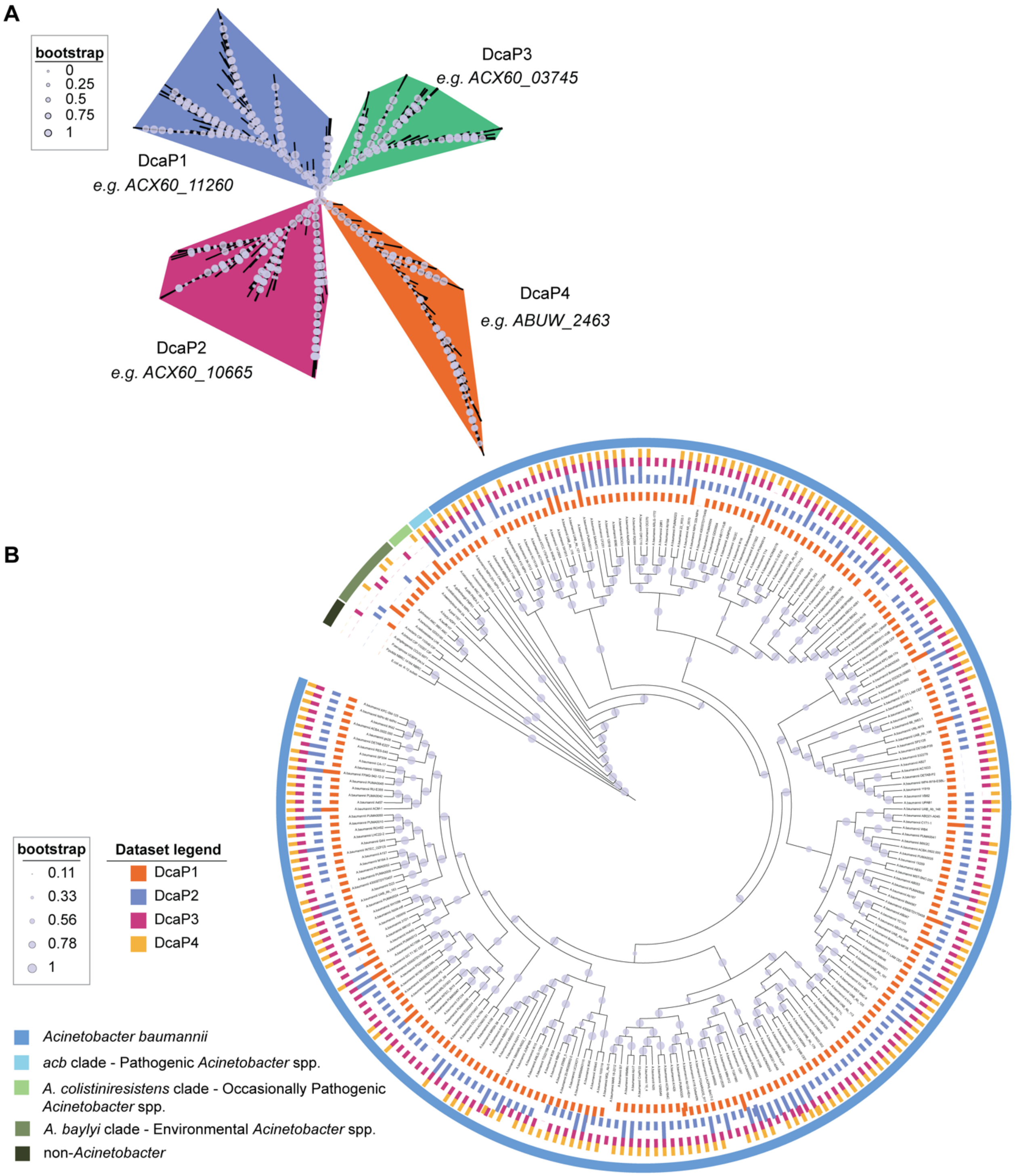

Figure S1. DcaP proteins are conserved across *Acinetobacter* (A) *A. baumannii* DcaP proteins clustering with bootstrap values displayed. **(B)** Phylogenetic tree of *Acinetobacter* strains and clades depicting prevalence of individual DcaP proteins. Color coded boxes indicate the presence, absence, or duplication of individual DcaP proteins. Clades within *Acinetobacter* are indicated by the outermost color bar circling the tree. The accession numbers of the data depicted are in Table S4.

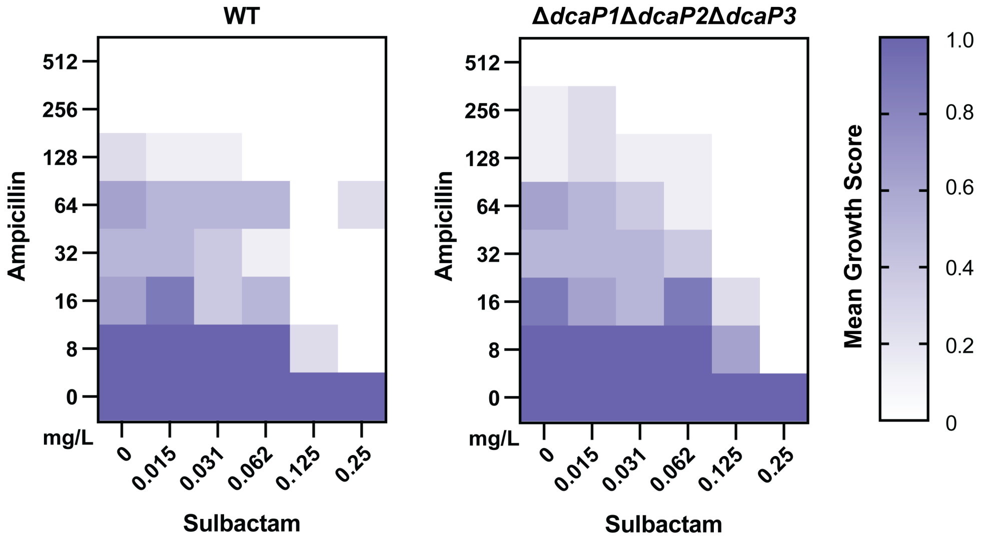

Figure S2. Checkerboard assay of ampicillin and sulbactam for wild-type and triple DcaP mutant. A 96-well plate was filled with stocks of LB with ampicillin and sulbactam and serially diluted to obtain 100 μL of unique concentrations for each compound in each well before inoculation with 1 μL of indicated bacterial strain. Growth was scored as 1, strong turbidity; 0.5, mild turbidity; 0, little to no turbidity. Experiment was repeated 3 times with an n=2 for a total n=6. Means are shown.

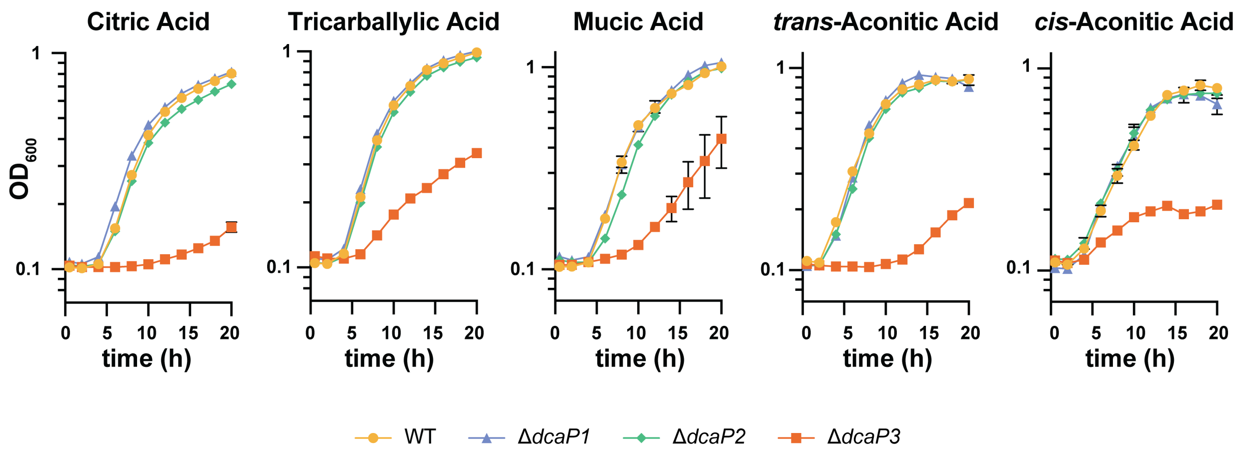

Figure S3. Growth curves of single Δ*dcaP* mutants on citric acid, tricarballylic acid, mucic acid, and *cis*- and *trans*- aconitic acids. Wild-type and single mutants lacking DcaP1, DcaP2, or DcaP3 in *A. baumannii* ATCC 17978 were grown in M9 media with the indicated compound as the sole carbon source. Data show mean ± SEM, n=3.

Table S1. Strains

Strain	Source	Identifier
A. baumannii ATCC 17978VU (WT)	ATCC	LP486
A. baumannii ATCC 17978VU *att*::mTn7(empty); Carb^{R}	Ren and Clark *et al.*, 2025 (1)	LP526
A. baumannii ATCC 17978 ∆*dcaP1::kan* (ACX60_11260)	This manuscript	LP1191
A. baumannii ATCC 17978 ∆*dcaP2::kan* (ACX60_10665)	This manuscript	LP1190
A. baumannii ATCC 17978 ∆*dcaP3::kan* (ACX60_03745)	This manuscript	LP1189
A. baumannii ATCC 17978 ∆*dcaP1*∆*dcaP2*::kan	This manuscript	LP1253
A. baumannii ATCC 17978 ∆*dcaP1*∆*dcaP3*∆*dcaP2*::kan	This manuscript	LP1308
A. baumannii ATCC 17978 ∆*dcaP3*∆*dcaP1*::kan	This manuscript	LP1302
A. baumannii ATCC 17978 ∆*dcaP3*∆*dcaP2*::kan	This manuscript	LP1227
A. baumannii ATCC 17978 ∆*dcaP3::kan, att::*mTn7(*dcaP3*)	This manuscript	LP1214
A. baumannii ATCC 17978VU *att*::mTn7(5075_*dcaPX*)	This manuscript	LP1314
A. baumannii AB5075	Colin Manoil (University of Washington)	LP345
A. baumannii AB5075 ∆*dcaP3*::hyg	This manuscript	LP1315

Table S2. Plasmids

Use or Phenotype	Source	Name
Kan^{R}; mobilization helper plasmid	Figurski and Helinski, 1979 (2)	pRK2013
Carb^{R}; plasmid encoding mTn7 transposition pathway	Choi *et al.*, 2005 (3)	pTNS2
Carb^{R}; allelic exchange vector with sucrose sensitivity	Hoang *et al.*, 1998 (4)	pFLP2
Contains an FRT-flanked kanamycin resistance gene	Datsenko and Wanner, 2000 (5)	pKD4
Contains a mTn7 with carbenicillin resistance gene	Carruthers *et al.*, 2013 (6) and Alexeyev, 1999 (7)	pKNOCK
Carb^{R}; construct to complement *dcaP3*	This manuscript	pKNOCK-*dcaP3*
Carb^{R}; express the AB5075 DcaP4 in ATCC 17978	This manuscript	pKNOCK-*dcaP4*
Carb^{R} Kan^{R}; deletion construct for DcaP1	This manuscript	pFLP2-*dcaP1*
Carb^{R} Kan^{R}; deletion construct for DcaP2	This manuscript	pFLP2-*dcaP2*
Carb^{R} Kan^{R}; deletion construct for DcaP3	This manuscript	pFLP2-*dcaP3*
Carb^{R} Hyg^{R}; deletion construct for DcaP3 in AB5075	This manuscript	pFLP2-5075*dcaP3*

Table S3. Oligonucleotides

All oligonucleotides were purchased from IDT (Coralville, IA)

Sequence	Identifier	Name
tgaacggcaggtatatgtgatggg	LP52	pFLP2 fwd
ccatgattacgaattcgagc	LP54	pFLP2 rev
gtgtaggctggagctgcttc	LP154	pKD4-FRTfrag_F
catatgaatatcctccttagttcctattc	LP155	pKD4-FRTfrag_R
ttcctttagcagcccttg	LP181	FRT_Frag_102_R
gatctcatgctggagttc	LP183	FRT_Frag_1384_F
cttctctcaaaataaggaacttcg	HN355	03745 up up fwd

caatccaagtcacttggtaac	HN356	03745 dn dn rev
aaaaggatcgatcctctagaggatcacatcaccatgcttggg	HN357	03745 up fwd
acttcgaagcagctccagcctacaccaaagactcctaaaaatatattttatgc	HN358	03745 up rev
aggaactaaggaggatattcatatgtaacgatttttttagctaaaaatagaaaag	HN359	03745 dn fwd
atgattacgaattcgagctcggtacagttgaaaatatcttgtttagaaagc	HN360	03745 dn rev
aaaaggatcgatcctctagaggatccgttgtatttaaaattttcatcatg	HN285	pFLP-11260-up-fwd
attctctagaaagtataggaacttctcaaaagctcggtcaaag	HN286	pFLP-11260-up-rev
aggaactaaggaggatattcatatgtcccgtttatttttctggtca	HN287	pFLP-11260-dn-fwd
atgattacgaattcgagctcggtacttcaacatgatagcgagttatg	HN288	pFLP-11260-dn-rev
gcaaagaacacatttcacac	HN318	11260 up up rev
ccagagcaagtatctaatcagac	HN319	11260 dn dn fwd
ctggtgcagaagatattgaag	HN351	10665_dcaP_up up fwd
cagacttcttaggtcgtgc	HN352	10665 dcaP dn dn rev
aaaaggatcgatcctctagaggatccaatgctgaagtagtgatgag	HN353	pFLP2-10665-up fwd
acttcgaagcagctccagcctacactggatataaaagatttccaatatgtgc	HN354	pFLP2-10665-up rev
aggaactaaggaggatattcatatgaatccggttaaaaatattgatttagg	HN363	pFLP2-10665-dn-fwd
atgattacgaattcgagctcggtacggtgtatttctgtagctcgt	HN364	pFLP2-10665-dn-rev
tcatgcatgagctcactagtggatcggttctgtaaaccaaaaagt	HN377	pKNOCK-03745_fwd
ggcctgcaaggccttcgcgaggtacttagaaactatatttagccattaaac	HN378	pKNOCK-03745_rev
tcatgcatgagctcactagtggatcgattggatattagcctaaagtct	HN400	dcaP3 promoter fwd
ctgcagctaaaattaatttttcatcaaagactcctaaaaatatattttatgc	HN401	dcaP3 promoter rev
taaaatatatttttaggagtctttgatgaaaaaattaattttagctgcag	HN402	dcaP4 fwd
ggcctgcaaggccttcgcgaggtacttagaatttatacatcgatacaaag	HN403	dcaP4 rev
aaaaggatcgatcctctagaggatcacatcaccatgcttgg	HN385	5075 dcaP3 up fwd
acttcgaagcagctccagcctacaccaaagactcctaaaaatatattttatg	HN386	5075 dcaP3 up rev
aggaactaaggaggatattcatatggaacgatttttagctaaaaatagaaaag	HN387	5075 dcaP3 dn fwd
atgattacgaattcgagctcggtacaaaagttgaaaatatcttgtttagaaag	HN388	5075 dcaP3 dn rev
gaacttcgttcttggttaatc	HN389	5075 dcaP3 up up fwd
ctttacaacccgagttattatc	HN390	5075 dcaP3 dn dn rev

Supplementary Methods

Checkerboard assay

In a 96-well plate, each well was filled with 100 µl LB. Stock solutions of ampicillin (100 mg/mL) and sulbactam (30 mg/mL) were diluted into LB for a final concentration of 1024 µg/mL and 32 µg/mL, respectively. An additional solution of 2048 µg/mL ampicillin was prepared. 100 µl aliquots of 1024 µg/mL ampicillin were placed in row A, wells 1-11, and 2024 µg/mL ampicillin in well 12. 2-fold serial dilutions were performed beginning in row A and ending in row H, discarding the remaining 100 µl. 100 µl aliquots of 32 µg/mL sulbactam were placed in every well in column 12, and 2-fold serial dilutions were performed beginning in column 12 and ending in column 2, discarding the remaining 100 µl. The maximum concentration for each antibiotic was therefore 512 µg/mL ampicillin and 16 µg/mL sulbactam. Then, 1 µl overnight culture of *A. baumannii* ATCC 17978 wild-type or the ΔΔΔ*dcaP* mutant was inoculated into each well before incubation at 37°C with shaking at 180 rpm for 8-16 hours. The following day, plates were scored for growth or no growth phenotypes. Wells with strong optical turbidity were assigned a value of ‘1’ and wells with mild turbidity or mild turbidity with clumped cells were assigned a value of ‘0.5’. Wells with no visual growth or fully clumped cells were assigned a value of ‘0.’

Supplementary References

1. Ren X, Clark RM, Bansah DA, Varner EN, Tiffany CR, Jaswal K, Geary JH, Todd OA, Winkelman JD, Friedman ES, Zemel BS, Wu GD, Zackular JP, DePas WH, Behnsen J, Palmer LD. 2024. Amino acid competition shapes *Acinetobacter baumannii* gut carriage. bioRxiv 2024.10.19.619093.

2. Figurski DH, Helinski DR. 1979. Replication of an origin-containing derivative of plasmid RK2 dependent on a plasmid function provided in trans. Proc Natl Acad Sci U S A 76:1648–1652.

3. Choi K-H, Gaynor JB, White KG, Lopez C, Bosio CM, Karkhoff-Schweizer RR, Schweizer HP. 2005. A Tn7-based broad-range bacterial cloning and expression system. Nat Methods 2:443–448.

4. Hoang TT, Karkhoff-Schweizer RR, Kutchma AJ, Schweizer HP. 1998. A broad-host-range Flp-FRT recombination system for site-specific excision of chromosomally-located DNA sequences: application for isolation of unmarked *Pseudomonas aeruginosa* mutants. Gene 212:77–86.

5. Datsenko KA, Wanner BL. 2000. One-step inactivation of chromosomal genes in *Escherichia coli* K-12 using PCR products. Proc Natl Acad Sci U S A 97:6640–6645.

6. Carruthers MD, Nicholson PA, Tracy EN, Jr RSM. 2013. *Acinetobacter baumannii* utilizes a type VI secretion system for bacterial competition. PLOS ONE 8:e59388.

7. Alexeyev MF. 1999. The pKNOCK series of broad-host-range mobilizable suicide vectors for gene knockout and targeted DNA insertion into the chromosome of gram-negative bacteria. Biotechniques 26:824–826, 828.
